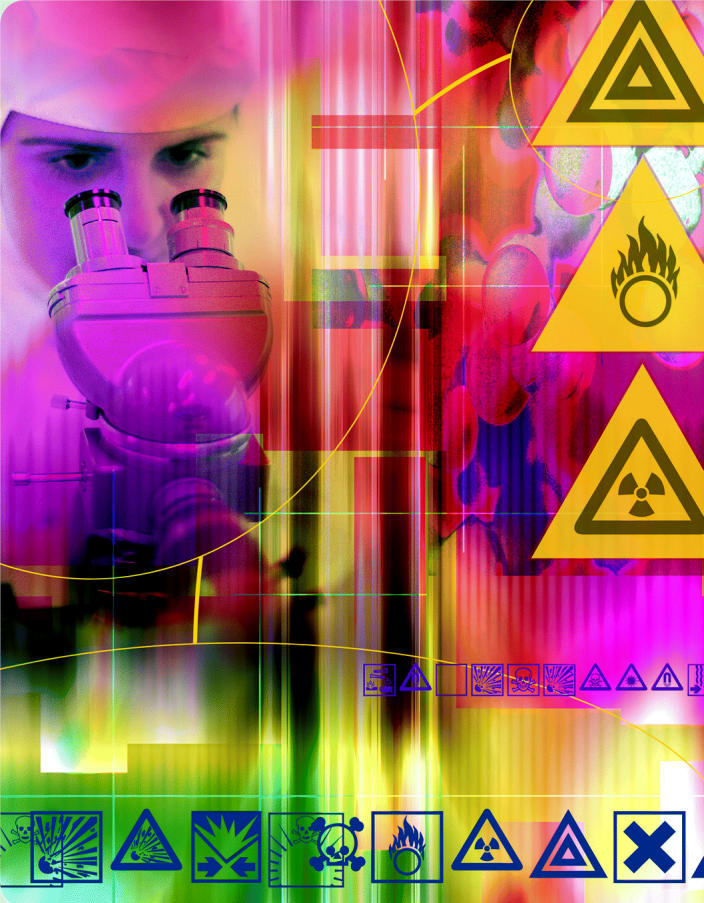# Development of Medical Countermeasures to Chemical Terrorism—The NIEHS’s Involvement in a Government-Wide Research Effort

**Published:** 2005-11

The attacks of September 11, 2001, using airplanes, followed closely by the biologic attacks using anthrax spores placed in letters, have illustrated how vulnerable our society is to such acts of terrorism. Since these events, the NIH has led the national research effort focused on the development of medical countermeasures to treat civilian mass casualties. The NIH has received more than $1.5 billion annually, starting in FY 2002, to fund research focused primarily on development of products to diagnose, treat, or prevent bacterial and viral infectious diseases and toxemias that could result from bioterrorist attacks. These initial efforts, focused on infectious agents, have been led by the National Institute of Allergy and Infectious Diseases. In FY 2005, the NIH was given an additional $47.1 million to establish a radiation and nuclear medical countermeasures program to identify products to treat civilians injured by a dirty bomb or nuclear attack. Similar plans are under way to initiate research in FY 2006 to develop products to diagnose and treat victims of a chemical attack.

As part of the planning for an expanded effort related to chemical agents, the NIEHS and the National Institute on Neurological Diseases and Stroke co-sponsored, in FY 2005, an administrative supplement program to existing grants. The intent of these supplements is to develop improved detection, diagnosis, and treatment strategies for likely chemical threat agents. As a result of this program, NIEHS has funded six supplements for a total of $450,000. The NIEHS supplements went to:

» Paul Bishop and Joseph Caruso, University of Cincinnati, to work on increasing the sensitivity of methods they have developed for detecting hydrolysis products of nerve agents and to extend their analysis to spiked food and water samples
» Brian Day, National Jewish Medical and Research Center, to test the ability of lipoic acid, dihydrolipoic acid, and thioredoxin to reverse or prevent pulmonary injury caused by sulfur mustard
» Richard DiGiulio and Ted Slotkin, Duke University, to study the developmental neurotoxicity of nerve agents and to assess some possible protective treatment strategies
» Clem Furlong, University of Washington, to attempt to increase the activity of human PON1 enzyme to levels sufficient to protect against paraoxon exposure in a mouse model. If successful, he will test the modified enzyme’s ability to protect against exposure to sarin, soman, and VX agents in the same mouse model.
» Bruce Hammock and Ian Kennedy, University of California, Davis, to work on the development of miniaturized sensors for use in detecting botulinum toxin, ricin, and abrin
» Cary Pope, Oklahoma State University, to study the differential toxicity of the organophosphorus pesticides chlorpyrifos and parathion, as well as the threat agents sarin and soman, and their inhibition of acetyl-cholinesterase and the accumulation of acetylcholine in a cannulated rat brain model

This administrative supplement program for selected grant mechanisms funded by NIEHS has just been reannounced for FY 2006 in the NIH Guide **(http://grants.nih.gov/grants/guide/notice-files/NOT-ES-06-001.html).**

## Contact

**Dennis Lang, PhD |** lang4@niehs.nih.gov

## Figures and Tables

**Figure:**